# Reducing firearm access for youth at risk for suicide in a pediatric emergency department

**DOI:** 10.3389/fpubh.2024.1352815

**Published:** 2024-05-22

**Authors:** Sofia Chaudhary, Kiesha Fraser Doh, Emilie Morris, Caroline Chivily, Donnetta S. Washington, Scott E. Gillespie, Andrew Jergel, Sarah Lazarus, Angela Costa, Nathan Call, Jonathan Rupp, Harold K. Simon

**Affiliations:** ^1^Department of Pediatrics, Emory University School of Medicine, Atlanta, GA, United States; ^2^Department of Emergency Medicine, Emory University School of Medicine, Atlanta, GA, United States; ^3^Children’s Healthcare of Atlanta, Atlanta, GA, United States; ^4^Department of Pediatrics, University of Utah, Salt Lake City, UT, United States; ^5^Emory University School of Medicine, Atlanta, GA, United States; ^6^School of Social Work, The University of GA, Athens, GA, United States; ^7^Pediatric Emergency Medicine Associates, Atlanta, GA, United States; ^8^Morehouse School of Medicine, Atlanta, GA, United States

**Keywords:** firearm, safe storage, lethal means, emergency department, pediatrics

## Abstract

**Background:**

Firearm-related suicide is the second leading cause of pediatric firearm death. Lethal means counseling (LMC) can improve firearm safe-storage practices for families with youth at risk of suicide.

**Objectives:**

This study aims to evaluate the feasibility of pediatric emergency department (ED) behavioral mental health (BMH) specialists providing LMC to caregivers of youth presenting with BMH complaints and to test for changes in firearm safety practices, pre-post ED LMC intervention, as measures of preliminary efficacy.

**Methods:**

Prospective pilot feasibility study of caregivers of youth presenting to a pediatric ED with BMH complaints. Caregivers completed an electronic survey regarding demographics and firearm safe-storage knowledge/practices followed by BMH specialist LMC. Firearm owners were offered a free lockbox and/or trigger lock. One-week follow-up surveys gathered self-reported data on firearm safety practices and intervention acceptability. One-month interviews with randomly sampled firearm owners collected additional firearm safety data. Primary outcomes were feasibility measures, including participant accrual/attrition and LMC intervention acceptability. Secondary outcomes included self-reported firearm safety practice changes. Feasibility benchmarks were manually tabulated, and Likert-scale acceptability responses were dichotomized to strongly agree/agree vs. neutral/disagree/strongly disagree. Descriptive statistics were used for univariate and paired data responses.

**Results:**

In total, 81 caregivers were approached; of which, 50 (81%) caregivers enrolled. A total of 44% reported having a firearm at home, 80% completed follow-up at one week. More than 80% affirmed that ED firearm safety education was useful and that the ED is an appropriate place for firearm safety discussions. In total, 58% of participants reported not having prior firearm safety education/counseling. Among firearm owners (*n* = 22), 18% reported rarely/never previously using a safe-storage device, and 59% of firearm owners requested safe storage devices.

At 1-week follow-up (*n* = 40), a greater proportion of caregivers self-reported asking about firearms before their child visited other homes (+28%). Among firearm owners that completed follow-up (*n* = 19), 100% reported storing all firearms locked at one week (+23% post-intervention). In total, 10 caregivers reported temporarily/permanently removing firearms from the home.

**Conclusion:**

It is feasible to provide LMC in the pediatric ED via BMH specialists to families of high-risk youth. Caregivers were receptive to LMC and reported finding this intervention useful, acceptable, and appropriate. Additionally, LMC and device distribution led to reported changes in safe storage practices.

## Introduction

Suicide is the second leading cause of death among the US youth aged 10–14 years and the third leading cause of death among those aged 15–24 years (1). From 2010–2021, 17,444 youth aged 10–17 years died by suicide, representing a 75.4% increase in suicide deaths over this period, with more than 40% resulting from firearms, the most lethal means (1, 2).

Access to household firearms is associated with an increased risk of youth suicide that can be modified with firearm safe storage (3–6). Currently, approximately 30 million US youth live in households with firearms, with 4.6 million (15%) of these homes reportedly storing at least one firearm, loaded and unlocked, the least safe method (7). The onset of the COVID-19 pandemic caused a significant increase in firearm sales, with more firearms being available and accessible in youth households (8–10). Both increased firearm access and the worsening mental health (MH) crisis during this period were significant risk factors for youth suicide (11, 12). From 2011 to 2020, there was a 5-fold increase in pediatric emergency department (ED) suicide-related visits, and from March to October 2020, ED MH-related visits increased by 24% for children aged 5–11 years and 31% for children aged 12–17 years compared to the year prior (11, 13). Furthermore, during the first year of the pandemic, there were significantly more suicides among younger children and minoritized youth, as well as more firearm suicides than expected (14). In October 2021, the American Academy of Pediatrics (AAP), American Academy of Child and Adolescent Psychiatry (AACAP), and Children’s Hospital Association (CHA) declared a national emergency in child MH (15). Soon after, the Surgeon General issued an advisory about the youth MH crisis (16). Pediatric EDs, often the first point of care for a child’s MH emergency, have seen the worsening MH crisis and increased suicide rates first-hand and necessitated an opportunity to intervene (11, 12, 17–19).

Lethal means counseling (LMC), where families are advised to reduce access to lethal means, including firearms and medications, is one suicide prevention strategy that has shown promising results in both adult and pediatric ED settings (20–24). One multi-center ED controlled trial that conducted an LMC intervention with the distribution of firearm storage devices for caregivers of at-risk youth found that twice as many caregivers improved firearm storage post-intervention (24). Similarly, a single pediatric hospital intervention study found that offering firearm storage devices along with training to household members of youth presenting with MH complaints improved firearm storage practices (23). Outside of these ED-based studies, firearm safety interventions in clinics and community settings that pair counseling with device distribution are the most effective in improving storage practices in contrast to offering to counsel alone (25–27).

This study aimed to explore the introduction of an LMC initiative in a tertiary southeastern United States (US) pediatric ED in a period of increasing rates of behavioral mental health (BMH) visits for suicidality. Our study objectives were to evaluate the feasibility and acceptability of pediatric ED BMH specialists providing LMC to caregivers of youth presenting with BMH complaints and to test for reported changes in firearm safety practices, pre-post ED LMC intervention, as measures of preliminary efficacy.

## Materials and methods

### Study design and data sources

We conducted a prospective pilot feasibility study of a convenience sample of parents and legal guardians (caregivers) of youth presenting to a pediatric emergency department with a BMH complaint between 14 January 2022 and 31 January 2023. Caregivers were enrolled in a 54-bed free-standing pediatric emergency department of a leading southeastern US tertiary pediatric healthcare system. This is a regional catchment hospital with an annual ED volume of 81,492 visits in 2022; of which, 1,821 (2.2%) were BMH encounters. This study was approved by the institutional review board at Emory University.

Participants were eligible for inclusion in the study if they were an English-speaking caregiver of a child of <18 years of age presenting with a BMH complaint or a BMH concern was raised during the ED visit, and the child was evaluated by an ED BMH specialist. Caregivers were eligible if the patient lived with them for any period during weeks/weekends.

Three ED BMH specialists (one licensed clinical social worker and two licensed professional counselors), each trained in LMC using the Counseling on Access to Lethal Means (CALM) online course detailed below, consented and enrolled caregivers for our study (20). These ED BMH specialists invited caregivers to participate in the study after providing their initial BMH assessment for the patient’s primary BMH concern(s). Enrollment occurred during their clinical shifts in an area separate from the child or teen patient whenever possible. Two of these three ED BMH specialists enrolled caregivers during a mix of weekday and weekend daytime hours (6:00 AM–6:30 PM) throughout the full study period; the third ED BMH specialist enrolled caregivers during nighttime hours (6:30 PM–6:00 AM), only during the last half (6 months) of the study period. All study consents were collected electronically via iPads and managed using Research Electronic Data Capture (REDCap) tools hosted at our institution. REDCap is a secure web-based software platform designed to support data capture for research studies (28, 29).

Study on-boarding and intervention were adapted for our study population based on a previous study by Miller and Salhi et al., including online training for ED BMH to facilitate LMC counseling, distribution of educational handouts and offsite storage handouts, and distribution of firearm safe storage devices (24, 30).

### Study on-boarding

Study ED BMH specialists all completed mandatory training up to 2 weeks before enrollment, including a free, 2 h, online course, CALM through Zero Suicide (20). Additionally, ED BMH specialists participated in a virtual study training session where the research team reviewed the scope of local pediatric firearm injuries and suicide risk and associated mortality data, study aims, an overview of the study LMC protocol, and types of firearm safe storage devices. The training session reiterated key points from the CALM course, including primarily recommending offsite firearm storage, especially when the child or teen is in a period of crisis. If the family reported being unable to store offsite, the recommendation was secure firearm storage, including storing all guns locked and unloaded with ammunition stored separately and locked. Additional recommendations included locking up all prescription medications (especially narcotics, sleep aids, and analgesics) except those that may be life-saving (20). Offsite firearm storage considerations and those of triple safe storage are consistent with recommendations from the recent American Academy of Pediatrics (AAP) policy statement and technical report (31, 32). Finally, all study ED BMH specialists were required to complete institutional CITI-training certification so they could enroll and consent patients for the study. This was done to minimize any disruptions in care for BMH patients. All ED BMH specialists were given a study outline document with sample conversation scripts for LMC and summarized key messages from CALM training, as detailed above, to have on hand during enrollment.

ED BMH specialists were selected to conduct the study intervention of LMC since they are the primary resource in our ED, and they routinely introduce the topic of restriction of lethal means in their assessment and during discharge planning of BMH patients. Partner meetings between ED, nursing, BMH, and social work leadership were conducted over a period of months before study implementation to discuss the need for the intervention, receive feedback, and develop an informed approach regarding study logistics. The study was also introduced at an ED division meeting before enrollment so that all ED team members were aware of the study and that there would be minimal disruptions to patient care or flow. Study investigators checked in monthly, either via meetings or email, with BMH specialists to identify and address any challenges or concerns with more frequent engagement in the first few weeks of study enrollment.

### Study intervention

#### Initial visit

During the ED visit, caregivers completed a self-administered 31-question baseline electronic survey developed and beta-tested by the investigative team via REDCap on a study iPad. This baseline survey questions were on self-reported demographics and reported firearm safe-storage knowledge and practices (Appendix A). After this initial questionnaire, ED BMH specialists provided a 5-min LMC intervention verbally and asked about the presence of firearm(s) in the home. Caregivers were advised to store all firearms away from home, even if only temporarily during crisis periods. If caregivers could not store offsite, they were advised to secure all firearms by triple safe storage, storing all firearms: (1) unloaded, (2) locked, and (3) with ammunition stored separately and locked to help reduce unauthorized access. If the caregiver endorsed having a firearm in the home, they were offered a free firearm safe storage device(s), a combination lock box [Bulldog (BD1126)], and/or a combination trigger lock [Bulldog (BD8000)]. The lock box retailed for $32.99, had a 3-digit combination code, a cable for anchoring, and could hold one handgun. The trigger lock retailed at $17.99, had a 3-digit combination code, and could be used on handguns or long guns. Caregivers were allowed to take up to one of each safe storage device depending upon their preference and type of firearm. These safe storage devices were chosen as they did not utilize a key that could potentially be found and were the same devices that were well-received by gun owners at our institutional community firearm safe storage events. Previous studies in community and ED settings suggest that lockboxes and gun safes are preferred but that device preference may vary according to gun type and gun purpose (24, 26, 33, 34). ED BMH specialists recorded responses in a baseline electronic survey for gun access at home, the type of safe storage device taken, and reasons for taking a particular type of device. If a trigger lock was taken, caregivers were still recommended to lock firearms in a firearm safe after the placement of the trigger lock. All caregivers were provided educational handouts, including an institutional firearm safety handout, a BeSMART handout discussing pediatric firearm suicide, and a local offsite firearm storage location handout developed by the study team (35). After receiving LMC, caregivers completed the final part of the baseline electronic survey that included reporting acceptability of the ED intervention and viewing a 30-s embedded video “#1 Secure” by BeSMART reiterating firearm secure storage recommendations (36). Patient demographic data (age and gender) and ED BMH-related discharge diagnoses were obtained by study investigators via chart review. ED BMH discharge diagnoses were categorized according to their *International Classification of Diseases, Tenth Revision* (ICD-10), diagnosis codes for either suicidal ideation (R45.851) or suicide attempt (T14.91XA) with all other remaining BMH ED discharge diagnoses grouped as other BMH issue.

#### Follow-up

All caregivers were emailed a link to the 1-week follow-up REDCap electronic survey (Appendix B) and copies of the educational handouts given in the ED. The survey gathered self-reported data on firearm safety practices and intervention acceptability. All participants who completed the one-week follow-up survey were emailed a $5 Amazon gift card for participation. Caregivers who reported gun ownership were invited to participate in a subsequent one-month follow-up 30-min Zoom interview with study team members by random sampling. Study team members conducting Zoom interviews completed the 2 h online CALM course and a training session with lead study investigators reviewing scripted messaging and motivational interviewing techniques before conducting interviews (20). These Zoom interviews were conducted to visualize how the caregiver was storing their firearm(s) post-intervention, provide feedback as needed, and obtain input regarding the ED intervention. All interview responses were recorded in REDCap. All participants who completed the 1-month Zoom follow-up were emailed an additional $5 Amazon gift card. For each of the 1-week and 1-month follow-up time points, the study team provided up to four reminders, each 2 days apart. Two reminders were automated via REDCap and two reminders were via the study team’s phone, text, or email.

### Study measures

#### Feasibility measures

Feasibility outcomes were as follows: (1) Accrual of participants, as measured by the acceptance rate [(number of accepted participants/number of approached participants) X 100] and percentage of the enrolled sample that was gun owning [(gun owning participants/number of enrolled participants) X 100]; (2) Attrition of participants, as evaluated by study completion rate (number of completing participants/number of enrolled participants) X 100; (3) Caregiver acceptability of the LMC intervention as indicated by a response of “Agree” or “Strongly Agree” on a two-item, 5-point Likert scale measure, indicating if study procedures were informative and the space appropriate. Barriers to the acceptability and feasibility of the intervention, as reported by ED BMH specialists, were monitored throughout the study period.

#### Efficacy measures

Preliminary efficacy outcomes included self-reported change in firearm safety practices. This included asking or planning to ask about guns in the home before child/teen drop off, frequency of safe storage device use, and storage practices of storing guns locked and unloaded, with ammunition stored separately. Questions regarding safe storage practices were adapted from the previous study by Simonetti et al. (26).

#### Statistical analysis

Feasibility benchmarks were manually tabulated. For acceptability feasibility measures, Likert-scale acceptability responses were dichotomized to strongly agree/agree (affirmative) vs. neutral/disagree/strongly disagree. For preliminary efficacy measures, descriptive statistics were used for univariate and paired data responses, and mean ± Standard Deviation (SD) was calculated where appropriate. Group comparisons between firearm owners and non-firearm owners were performed using Pearson’s Chi-squared test (Fisher’s exact test where appropriate) for categorical variables and two-sample *t*-test for continuous variables. Several variables were collapsed for statistical testing only. First, those with “Prefer not to say” were marked as NA (only in the variables gender, ethnicity, and race). Second, the location of firearm counseling collapsed as follows: ED/Doctor vs. Community Event/Police Department/Family/Friends vs. Other (includes gun shop/place of purchase, website, and others). The *p*-values of less than 0.05 were considered statistically significant. The absolute and relative change was calculated to determine self-reported practice changes overall and among firearm owners and non-firearm owners. All data cleaning and statistical testing were performed in R Statistical Software (v4.2.1; R Core Team 2022).

## Results

### Sample characteristics

In total, 50 caregivers enrolled in the study. The majority of caregivers were female (96%), Black (52%), non-Hispanic (76%), with a mean age of 40 (±8.12), and cared for 3 or more children regularly (52%) (Table 1). Among them, 22 endorsed having a firearm in their home. The mean age of the child presenting with the caregiver at the ED was 13 (± 2.66), and 62% were female. In total, 54% of presenting youth had a primary ED discharge diagnosis of suicidality (either suicidal ideation or suicide attempt).

**Table 1:** Participant demographics and characteristics.

Caregiver characteristics	Overall N = 50^a^	No firearm at home *N* = 28^a^	Firearm(s) at home *N* = 22^a^	*p*-value^b^
*Gender*				0.189^†^
Female	48 (96%)	28 (100%)	20 (91%)	
Male	2 (4%)	0 (0%)	2 (9%)	
Prefer not to say	0 (0%)	0 (0%)	0 (0%)	
*Ethnicity*				0.254^‡^
Hispanic or Latino	3 (6%)	3 (11%)	0 (0%)	
Non-Hispanic or Latino	38 (76%)	21 (75%)	17 (77%)	
Other	5 (10%)	1 (4%)	4 (18%)	
Prefer not to say	4 (8%)	3 (11%)	1 (5%)	
*Race*				0.102^‡‡^
Black or African American	26 (52%)	17 (61%)	9 (41%)	
White	15 (30%)	5 (18%)	10 (45%)	
Asian	1 (2%)	0 (0%)	1 (5%)	
American Indian or Alaskan Native	0 (0%)	0 (0%)	0 (0%)	
Native Hawaiian or Pacific Islander	0 (0%)	0 (0%)	0 (0%)	
Other	1 (2%)	1 (4%)	0 (0%)	
Prefer not to say	7 (14%)	5 (18%)	2 (9%)	
*Age*	39.70 (8.12)	39.50 (8.50)	39.95 (7.80)	0.845
*Child’s age*	12.70 (2.66)	12.29 (2.64)	13.23 (2.65)	0.218
*Child’s gender*				0.833
Female	31 (62%)	17 (61%)	14 (64%)	
Male	19 (38%)	11 (39%)	8 (36%)	
*How many children live in your home or do you care for regularly?*				0.798
1	8 (16%)	4 (14%)	4 (18%)	
2	16 (32%)	10 (36%)	6 (27%)	
3 or more	26 (52%)	14 (50%)	12 (55%)	
Age ranges of children at home*				
0–4 years	20 (40%)	11 (39%)	9 (41%)	0.754
5–9 years	21 (42%)	13 (46%)	8 (36%)	0.474
10–14 years	41 (82%)	23 (82%)	18 (82%)	>0.999
15–17 years	19 (38%)	7 (25%)	12 (55%)	0.033
*Child’s ED discharge diagnosis*				0.349
Suicidal ideation	23 (46%)	15 (54%)	8 (36%)	
Suicide attempt	4 (8%)	1 (4%)	3 (14%)	
Other behavioral mental health issues	23 (46%)	12 (43%)	11 (50%)	

a *n* (%); Mean (SD).

b Fisher’s exact test; Welch Two-Sample t-test; Pearson’s Chi-squared test.

*Multiple responses allowed. †*p*-value is derived from the comparison: male vs. female. ‡*p*-value is derived from the comparison: Hispanic/Latino vs. Non-Hispanic/Latino. ‡‡*p*-value is derived from the comparison: White vs. Black/AA vs. Other (Categories “Asian” and “Other” with only one individual each were combined into Other).

There were no statistically significant differences between participant demographics and baseline characteristics when comparing non-firearm owners and firearm owner caregivers except for the age ranges of children cared for within the home. Firearm owner caregivers cared for a higher percentage of 15–17-year-olds within the home (*p* = 0.033).

### Feasibility outcomes

**Figure 1:**
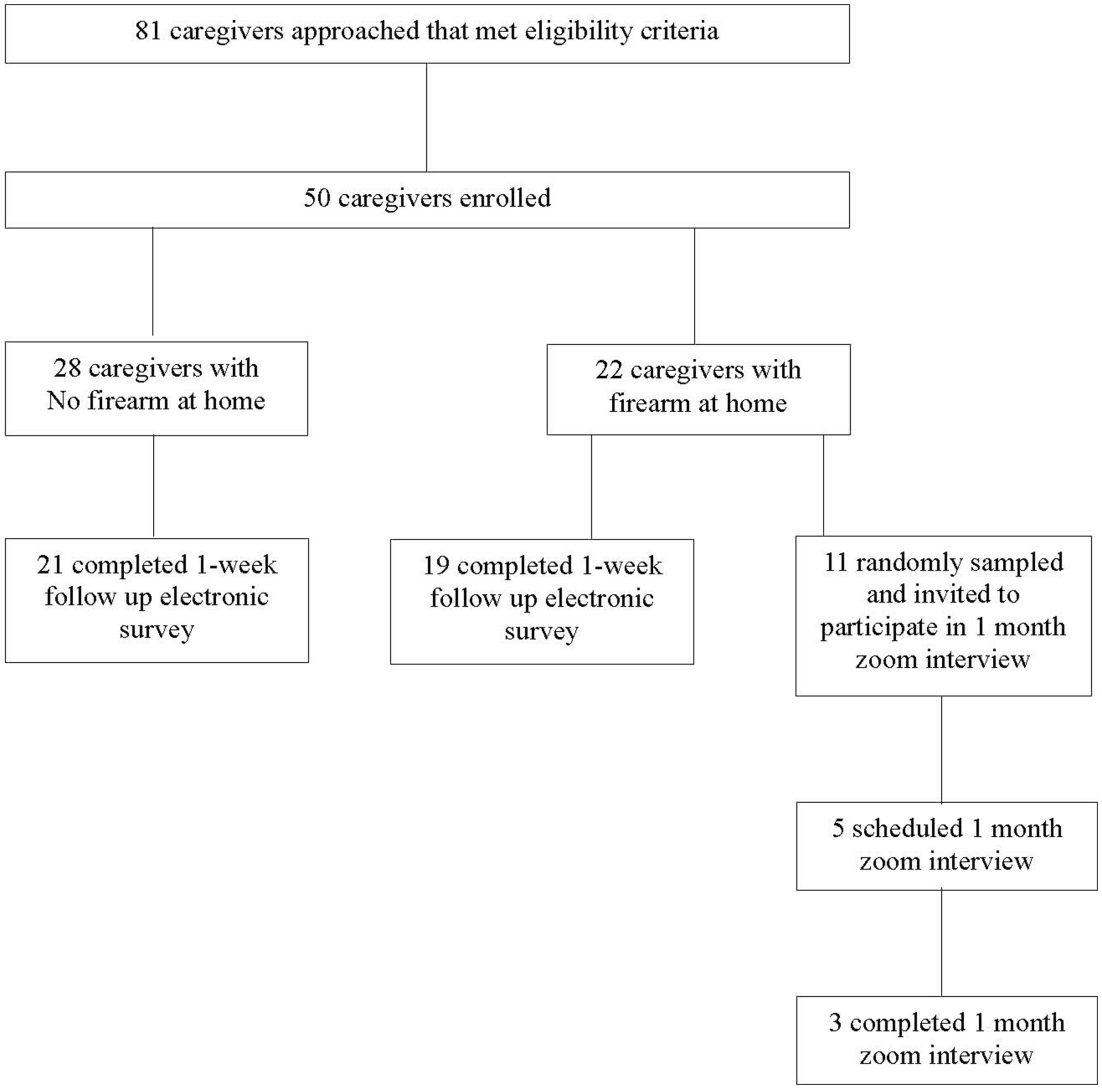
Study enrollment and follow up. Caregivers are parents/legal guardians of a child or teen who presented to the pediatric emergency department with a behavioral mental health complaint.

For accrual, there was an 81% acceptance rate with 50 out of the 62 caregivers approached that met eligibility criteria enrolling (Figure 1). In total, 44% (*n* = 50) of caregivers reported having a firearm at home (Table 2). For attrition, 80% of participants who completed the initial ED intake survey completed the 1-week follow-up; while 28% of randomly sampled firearm owners (*n* = 11) completed the 1-month Zoom follow-up. For acceptability, more than 80% of participants agreed both immediately after the ED education intervention and 1 week later that the education given was useful and appropriate in the ED setting. For procedural fidelity, protocol deviations were low overall (*n* = 3 out of 50 patient encounters). For the ability to manage the study and implement the intervention, there were no reported disruptions to patient care or flow. Study check-ins with ED BMH specialists throughout the study period revealed barriers such as technical challenges with the iPad during the ED caregiver survey and the length of time for the study consent process. ED BMH specialists reported the following facilitators for conducting the intervention: pre-intervention training, taking the CALM course training, and the ability to provide immediate resources (educational material and devices) in hand during the LMC conversation.

**Table 2:** Feasibility benchmarks.

Domain	Benchmark	Actual
*Accrual*		
All participants	>50% acceptance	81% (50/62)
Firearm owners	25–50% of study sample	44% (22/50)
*Attrition*		
Follow-up at 1 week (All)	>50% complete pre/post survey	80% (40/50) (All) 75% (21/28) among non-firearm owners 86% (19/22) among firearm-owners
Follow-up at 1 month (Firearm owner random sample)	>50% complete post zoom interview	28% (3/11)
*Acceptability*		
Informative	>75% agree/strongly agree	90% (45/50) immediately after ED intervention 85% (34/40) at 1 week follow-up
Appropriate place	>75% agree/strongly agree	84% (42/50) immediately after ED intervention. 85% (34/40) at 1 week follow-up

### Self-reported baseline firearm safety knowledge and practices

In total, 29 of all caregivers (58%, *n* = 50) and 11 of the caregivers with a firearm(s) at home (45%, *n* = 22) reported having not received prior education or counseling on firearm safe storage (Table 3). Only 12% of all caregivers reported having received prior firearm safety counseling from their child’s doctor’s office and 6% from their child’s prior ED visits. At baseline, 58% of all caregivers and 45% of caregivers with a firearm(s) at home reported not asking about firearms before their child/teen visited other homes. There were no significant differences between non-firearm owner caregivers and firearm owner caregivers for baseline firearm safety knowledge and the practice of asking about firearms in the home.

**Table 3:** Baseline caregiver firearm safety knowledge and firearm access.

Characteristic	Overall, *N* = 50^a^	No Firearm at Home, *N* = 28^a^	Firearm(s) at Home, *N* = 22^a^	*p*-value*^b^*
*Received prior firearm safety counseling/education*	21 (42%)	9 (32%)	12 (55%)	0.111
Location of Counseling*				0.286^†^
Doctor’s office- child	6 (12%)	3 (11%)	3 (14%)	
Doctor’s office- parent	0 (0%)	0 (0%)	0 (0%)	
Prior ED visit- child	3 (6%)	1 (4%)	2 (9%)	
Prior ED visit- parent	1 (2%)	1 (4%)	0 (0%)	
Community event	3 (6%)	2 (7%)	1 (5%)	
Police Department	2 (4%)	1 (4%)	1 (5%)	
Family, relative, or friend	4 (8%)	3 (11%)	1 (5%)	
Gun shop or place of purchase	4 (8%)	1 (4%)	3 (14%)	
Website	3 (6%)	2 (7%)	1 (5%)	
Other	5 (10%)	1 (4%)	4 (18%)	
*Child visits other homes with firearms*	15 (30%)	6 (21%)	9 (41%)	0.136
Knowledge of how these firearms are stored	8 (53%)	2 (33%)	6 (67%)	0.315
*Ask about firearms prior to the child visiting other homes*	21 (42%)	9 (32%)	12 (55%)	0.111
*If no, reasons for not asking:*				0.125
Never thought about asking	20 (69%)	13 (68%)	7 (70%)	
The child does not go on play dates or to other homes	7 (24%)	6 (32%)	1 (10%)	
A family member or someone they trust	2 (7%)	0 (0%)	2 (20%)	

a *n* (%). ^b^Fisher’s exact test; Welch Two Sample t-test; Pearson’s Chi-squared test.

*Multiple responses allowed. †*p*-value is derived from the comparison: ED/Doctor vs. Community Event/Police Department/Family/Friends vs. Other (includes gun shop/place of purchase, website, and other).

Among firearm owners (*n* = 22), 59% reported having one firearm at home; however, one-third of firearm owners reported having 3 or more guns at home (Table 4). Handguns (*n* = 18) were the most common type of firearm owned, followed by a shotgun (*n* = 9). Gun safes were the most commonly used type of safe storage device, followed by gun lock boxes. In total, 64% of firearm owners reported always using a safe storage device, while 14% never used one. A total of 77% of firearm owners reported storing all firearms currently locked, 72% storing firearms unloaded, and 77% with ammunition stored separately. Approximately one-third of firearm owners reported having stored firearms outside of their home when their child has been in a period of crisis.

**Table 4:** Baseline caregiver self-reported firearm storage practices.

Characteristic	Firearm at home, *N* = 22^a^
*How many firearms are in the home?*	
1	13 (59%)
2	2 (9%)
3 or more	7 (32%)
Location of firearm storage*	
Within the living spaces (including the bedroom)	13 (59%)
Basement	4 (18%)
Attics	3 (14%)
Garage/Shed	4 (18%)
Car	0 (0%)
Other	10 (45%)
Types of firearms*	
Handguns (including revolvers and pistols)	18 (82%)
Rifles	6 (27%)
Shotguns	9 (41%)
Assault/Military-style weapons (example AR 15)	0 (0%)
Other	0 (0%)
*Purpose of firearm*	
Safety/Protection	15 (68%)
Recreational (sport, hunting, or shooting range)	4 (18%)
Job: Armed forces, Law enforcement, Security	2 (9%)
Display/Decoration	0 (0%)
Family Heirloom	1 (5%)
Other	0 (0%)
Type of safe storage device*	
None	4 (18%)
Trigger lock	5 (23%)
Cable lock	5 (23%)
Gun lockbox	7 (32%)
Gun safe	10 (45%)
Other	1 (5%)
*How often are safe storage devices used?*	
Always	14 (64%)
Most of the time	2 (9%)
Sometimes	2 (9%)
Rarely	1 (4%)
Never	3 (14%)
*Firearms currently stored locked at home*	
Yes, all are stored locked	17 (77%)
Yes, some are stored locked	1 (5%)
None of the guns are stored locked	3 (14%)
I do not know	1 (5%)
*Firearms stored unloaded*	16 (72%)
*Ammunition stored separate from the firearm*	17 (77%)
*Firearm ever stored outside of the home if the child is in crisis*	7 (32%)
*Requested safety device*	13 (59%)
*The main reason for the device request*	
To keep people in my home safe	12 (92%)
To keep guns from being stolen	1 (8%)
To store other valuables (not guns)	0 (0%)
*Plan to immediately use the device*	13 (100%)

a *n* (%).

^*^Indicates multiple responses allowed.

### Study intervention

In total, 13 firearm owners (59%, *n* = 22) requested a study safe storage device (Table 4). Among these firearm owners who requested a study safe storage device, 62% reported at baseline storing all firearms locked and 69% reported storing firearms unloaded. In comparison, for firearm owners who did not request a study device (*n* = 9), 100% reported at baseline storing all firearms locked and 78% storing firearms unloaded. In total, 15 safe storage devices were distributed over the course of the study, including 10 lock boxes and 5 trigger locks (Table 5).

**Table 5:** Baseline caregiver firearm storage practices, BMH specialist verbal interview.

Characteristics	*N* = 22^a^
Firearms in the home	22 (100%)
Request firearm safe storage device	13 (59%)
Reason for declining device	
*Already have a device*	9 (41%)
*Prefer a different device than what was offered*	1 (5%)
*Do not want to lock up their gun*	0 (0%)
Lockbox requested	10 (77%)
Trigger lock requested	5 (38%)

a *n* (%).

### Preliminary efficacy outcomes

At the 1-week follow-up, a greater proportion of all caregivers (+28%) and a greater proportion of firearm owners (+13%) self-reported that they had asked about firearms in the home before their child/teen visited other homes (Table 6). Overall, more than 85% of caregivers self-reported at baseline and 1-week post-study intervention that they will inquire in the future about firearms in other homes before their child/teen visits. Gun safety information from the ED intervention was reported to be shared with others by 45% of all caregivers (*n* = 40) in follow-up, including 74% of firearm (*n* = 19) owners. Changes in firearm storage practices at follow-up included a greater proportion of firearm owners reported storing all firearms currently locked (+23%). In total, 10 firearm owner caregivers self-reported in follow-up that they have removed firearms either temporarily or permanently from their homes. These firearm owners stated that keeping their children safe was the most common reason for removing their firearms. Among these firearm owners who reported removing a firearm from their home (*n* = 10), at baseline, 60% stored all firearms locked and 70% stored firearms unloaded. In comparison, for firearm owners who did not remove their firearms from their homes (*n* = 12), at the baseline, 89% stored all firearms locked and 70% stored firearms unloaded.

**Table 6:** Caregiver self-reported practice changes.

Characteristic (All caregivers)	Initial ED Encounter *N* = 50^a^	1-Week Follow-Up, *n* = 40^a^	Absolute Change (%)	Relative Change (%)
Currently asks about firearms in other homes	21 (42%)	28 (70%)	28	67
Will ask about firearms in other homes*	46 (92%)	35 (88%)	−4	−4
Firearm safety education useful*	45 (90%)	34 (85%)	−5	−6
ED appropriate location to discuss firearm safety*	42 (84%)	34 (85%)	1	1

Characteristic (No firearm at home)	Initial ED Encounter *N* = 28^a^	1-Week Follow-Up, *n* = 21^a^	Absolute Change (%)	Relative Change (%)
Currently asks about firearms in other homes	9 (32%)	15 (71%)	39	122
Will ask about firearms in other homes*	26 (93%)	18 (86%)	−7	−8
Firearm safety education useful*	27 (96%)	19 (90%)	−6	−6
ED appropriate location to discuss firearm safety*	23 (82%)	18 (86%)	4	5

Characteristic (Firearm(s) at home)	Initial ED Encounter *N* = 22^a^	1-Week Follow-Up, *n* = 19^a^	Absolute Change (%)	Relative Change (%)
Currently asks about firearms in other homes	12 (55%)	13 (68%)	13	24
Will ask about firearms in other homes*	20 (91%)	17 (89%)	−2	−2
Firearm safety education useful*	18 (82%)	15 (79%)	−3	−4
ED appropriate location to discuss firearm safety*	19 (86%)	16 (84%)	−2	−2
Frequency of using a safe storage device
*Always*	14 (64%)	15 (79%)	15	23
*Most of the time*	2 (9%)	3 (16%)	7	78
*Sometimes*	2 (9%)	0 (0%)	−9	−100
*Rarely*	1 (4%)	1 (4%)	0	0
*Never*	3 (14%)	0 (0%)	−14	−100
Guns currently locked				
*Yes, all are stored locked*	17 (77%)	19 (100%)	23	30
*Yes, some are stored locked*	1 (5%)	0 (0%)	−5	−100
*None of the guns are stored locked*	3 (14%)	0 (0%)	−14	−100
*I do not know*	1 (5%)	0 (0%)	−5	−100
Firearms stored unloaded	16 (73%)	14 (74%)	1	1
Ammunition stored separately from the firearm	17 (77%)	16 (84%)	7	9

*Post ED Education and Likert Scale responses for agreed or strongly agreed.

^a^*n* (%).

Three firearm owner participants completed the 1-month follow-up Zoom interview (Table 2; Figure 1). One of the three participants showed the study team where and how they stored their firearms at home via Zoom. Two of the three had taken a study lockbox and were currently using it to store their firearm locked. When asked what type of safe storage device we should provide to families, all three participants primarily recommended lockboxes.

## Discussion

In this pilot feasibility study, we found that caregivers of youth presenting with acute BMH complaints were receptive to receiving an LMC intervention. There were self-reported improvements in caregiver firearm safety and storage practices post-intervention. To the best of our knowledge, this is one of the first studies after the onset of the COVID-19 pandemic evaluating an LMC intervention in a Southeastern United States high-volume pediatric ED. This was done in a period of increasing risk from acute BMH crisis and firearm(s) ownership. Given our findings and that of prior work, we believe a brief LMC intervention with the provision of firearm safety devices should be offered routinely for families presenting with youth at risk for suicide (21, 23, 24, 37).

Most (81%) of our caregivers who were approached and met eligibility criteria received LMC from our ED BMH specialists, illustrating the feasibility of this intervention in our pediatric ED. Study onboarding, training, and divisional/institutional buy-in were critical for the success of enrollment. CALM online training and study scripted messaging utilized in our study were reported by our ED BMH specialists to be accessible and to improve their comfort and self-efficacy in approaching families and delivering the LMC intervention uniformly (20). This is consistent with a prior community-based mental healthcare worker survey demonstrating that those who did the CALM training had increased comfort in and rates of providing LMC (38). Similarly, previous studies in two single-center high-volume sites in different regions of the US, Mueller et al. reported in an adult academic ED utilizing the CALM course and Runyan et al. reported in a pediatric ED utilizing online training based on CALM principles, also found their LMC interventions feasible with 77 and 81% enrollment rates, respectively (21, 22). Our study utilized ED BMH specialists, given their knowledge, expertise, and 24-h coverage for our ED BMH patients. Although behavioral health providers are more likely to ask about the presence of firearms in comparison to ED providers, this staffing model may not be available to all emergency departments (39). Brief online LMC training, such as CALM, or adapted training, such as those in the studies cited above, could provide critical resources for emergency departments to scale up LMC via ED physician providers or other appropriate clinical staff. Furthermore, emergency departments with written standard practice guidelines or protocols for discharge safety planning have been shown to have higher rates of LMC for all suicidal patients (40).

Caregivers found our ED LMC intervention during their child’s acute MH visit acceptable and appropriate. This supports the findings by Mueller, Runyan, and colleagues in their ED LMC intervention studies (21, 22). In our study, ED BMH specialists addressing the patient’s primary BMH concern first may have built trust and receptivity among caregivers for the LMC intervention. Second, the LMC intervention was designed with nursing, BMH, and ED partner input to reduce disruptions to routine patient care and flow. Caregiver receptivity did not change when moving from only daytime enrollment to daytime and overnight enrollment. Despite technology barriers, we could still enroll a convenience sample of caregivers. Reporting of firearm access was consistent between the initial ED survey caregiver self-reporting and subsequent verbal reporting with ED BMH specialist LMC conversation. Additionally, the majority of our firearm owners were receptive to taking safe storage devices. Our study’s in-service session, focusing on training for safe storage devices, was reported to increase the comfort of BMH specialists in device distribution and may have contributed to caregiver receptivity. A prior study comparing BH provider vs. ED provider LMC practices found that most of the participants in either group did not believe they had received enough training regarding firearm safe storage devices and that training would help them support patients in firearm access and storage decisions (39).

We had a good overall response rate (81%) to electronic 1-week follow-up surveys, with rates being high for both firearm owner and non-firearm owner subgroups. Gift card remuneration and the flexibility of doing surveys on their own time via email or text link may have incentivized participants. Response rates (28%) for 1-month Zoom interviews among a subgroup of firearm owners were much lower. There were some technical challenges with connectivity over Zoom, and some were either lost to follow-up after scheduling an interview date or non-responders. The requirement of being at home for the study team to visualize and validate self-reporting of firearm storage may have been challenging for participation. Additionally, phone or computer/tablet access for Zoom participation may not have been possible for all caregivers. This is, however, to the best of our knowledge, the first study that has utilized Zoom interviews to try to confirm firearm storage practices. As this was an exploratory measure in our study, future work should involve caregiver firearm owner input on the best ways to optimize this type of interview.

Overall, self-reported firearm safety practices improved from the initial ED LMC intervention to the 1-week follow-up. This demonstrates that it is possible to get positive reported behavior change with LMC education and the distribution of free firearm safe storage devices. This is consistent with the work from prior single-center ED studies and a multi-center controlled ED trial (21, 23, 24). In our study, firearm owners who responded to follow-up reported storing all firearms locked. This is encouraging as Monteaux et al. found that even modest adaptations of locking all household firearms could result in significant reductions in youth firearm suicides (6). More families were also asking or planning to ask about firearms and how they are stored before their child visits other homes after our intervention. Prior work in our population has shown that a majority of children are unable to recognize the difference between a toy gun and a real gun; thus, this question could be life-saving (41). Unexpectedly, 10 families reported either temporarily or permanently removing firearms from their homes after our ED LMC intervention. The risk of youth suicide is increased by 2-5x when firearms are present in the home, and reducing ready access to this most lethal means of suicide with barriers, such as offsite storage, can increase chances of survival (42–44). Key messaging from the CALM course of offsite storage is that storing firearms offsite is the safest while the child/teen is in a crisis period, and the delivery of messaging by our BMH specialists may have facilitated this reported caregiver behavior.

### Limitations

There are several cautions when interpreting the findings of our study. First, the outcomes were self-reported and susceptible to recall and social desirability bias as participants may have misremembered or not been forthcoming regarding firearm ownership and firearm safety practices. Second, the small sample size and data being collected from a single center in the Southeastern US may not be generalizable to other populations. However, this is a region with both increasing suicide and firearm ownership rates. Third, while this was a convenience sample and potentially not reflective of firearm ownership rates, the reported gun ownership of 44% is only slightly lower than our state firearm ownership rates. Fourth, caregiver participants who reported access to firearms at home may not have been the primary firearm owners. Given our low video follow-up rates among firearm owners, we were unable to fully validate self-reporting. Fifth, being a convenience sample of participants identified by ED BMH specialists, the population enrolled may not be reflective of all caregivers of youth at risk for suicide. Sixth, our center had 24 h BMH specialist coverage and thus cannot determine if our training and implementation of LMC intervention would have the same results in emergency departments with different staffing models. However, the CALM program can be used and adopted by non-BMH providers. Seventh, we did not further categorize the BMH concern for which the child was diagnosed outside of suicidal thoughts or suicide attempts. Future work should evaluate the different types of BMH issues that youth are presenting with to better understand the population. Given that children with mental health disorders are at increased risk of suicide, we had our ED BMH specialists include all BMH complaints for the LMC intervention and not only those presenting with active suicidal thoughts or after a suicide attempt (45, 46). Finally, caregiver inclusion was limited to English speakers only, as our educational materials were not available in other languages.

## Conclusion

Our study, one of the first in the Southeastern US, a high-risk region for both BMH concerns and firearm ownership, suggests that a brief lethal means counseling intervention for caregivers of youth at risk of suicide is a feasible and acceptable measure and resulting in reported positive behavior changes. As we continue to see increasing rates of ED youth MH visits for suicidality, increased accessibility of firearms, and escalating rates of youth firearm suicide, this intervention is promising (1, 8, 11, 12). Furthermore, brief suicide interventions in acute care settings are associated with decreased repeat suicide attempts and increased MH care follow-up (47). Future efforts of our study team will include scaling our LMC intervention for widespread implementation among all ED BMH visits at each of our three pediatric ED sites in our tertiary healthcare system. Continued research is needed to assess the longitudinal impacts of these ED-based interventions in different populations with a variety of ED staffing models to help provide a framework for best practices.

## Data availability statement

The original contributions presented in the study are included in the article/Supplementary material, further inquiries can be directed to the corresponding author.

## Ethics statement

The studies involving humans were approved by Emory University Institutional Review Board. The studies were conducted in accordance with the local legislation and institutional requirements. Written informed consent for participation in this study was provided by the participants’ legal guardians/next of kin.

## Author contributions

SC: Conceptualization, Data curation, Funding acquisition, Investigation, Methodology, Project administration, Supervision, Writing – original draft, Writing – review & editing. KD: Conceptualization, Writing – review & editing. EM: Investigation, Project administration, Writing – review & editing. CC: Investigation, Project administration, Writing – review & editing. DW: Investigation, Writing – review & editing. SG: Formal analysis, Writing – review & editing. AJ: Formal analysis, Writing – review & editing. SL: Writing – review & editing. AC: Writing – review & editing. NC: Writing – review & editing. JR: Conceptualization, Supervision, Writing – review & editing. HS: Conceptualization, Resources, Supervision, Writing – review & editing.
