# Supplementary material for: Reducing firearm access for youth at risk for suicide in a pediatric emergency department
Source: Front Public Health. 2024 May 22;12:1352815. doi: 10.3389/fpubh.2024.1352815 (PMC11163968; doi:10.3389/fpubh.2024.1352815)
Supplement: Supplementary file 1 [file Data_Sheet_1.PDF]

Caregiver Intake Survey Tool:

Demographics and characteristics (all participants)

1. What is your gender?
   - Female
   - Male
   - Transgender
   - Other, please state here_________
   - Prefer not to say
2. What is your ethnicity?
   - Hispanic or Latino
   - Not Hispanic or Latino
   - Other, please state here__________
   - Prefer not to say
3. What is your race?
   - White
   - Black or African American
   - Asian
   - American Indian or Alaskan Native
   - Native Hawaiian or Other Pacific Islander
   - Other, please state here_________
   - Prefer not to say
4. What is your age in years? __________
5. How many children under the age of 18 live in your home or do you care for regularly?

   ____

   (number of children)
6. What are the age ranges of the children that either live in your home or that you care for regularly? (Select all that apply)
   - < 1 year
   - 1-4 years
   - 5-9 years
   - 10-14 years
   - 15-17 years
7. Have you received gun safety/gun storage education or gun safety counseling in the past?
   - Yes
   - No

   If yes, where you have received gun safety/gun storage education or counseling? (Select all that apply)
   - Your child's pediatrician's office or doctor's office
   - Your doctor's office
   - Prior emergency department visit for child
   - Prior emergency department visit for myself
   - Community event
   - Police department
   - Family member, relative, or friend

- o Gun shop or place where I purchased a firearm
- o Website
- o Other, please list here______

Current Practices (all participants)

8. Does your child ever visit other people that have guns within their home (such as a relative, friend, or community member)?
   - o Yes
   - o No

   If yes, do you know how they are stored?
   - o Yes
   - o No
9. Before your child goes for a playdate or before your teenager visits a new place: Do you ask about guns being present (example: friend's or relatives' home)?
   - o Yes
   - o No

   If not, why not?
   - o I've never thought about asking
   - o I'm too uncomfortable to ask
   - o It was a family member or someone I trust
   - o My child doesn't go on play dates or to other people's homes
   - o Other, please state other reason here_______
10. Is there ever a gun present in your home?
    - o Yes
    - o No

    If yes, does your child or teen know where you keep your gun?
    - o Yes
    - o No

    If yes, has your child or teen ever touched or handled your gun to your knowledge?
    - o Yes
    - o No
11. At any time is a gun brought by somebody else into your home?
    - o Yes
    - o No

    If yes, what do you do if someone brings a gun to your home?
    - o Take no action
    - o Tell them they cannot enter with the gun
    - o Lock it up or store it out of reach from children
    - o Other, please state here_____________

***Caregivers with gun present at home* only**

12. How many guns do you currently have at home (including living spaces, basement, garage/sheds, or attics)?
    - o 0
    - o 1
    - o 2
    - o 3 or more
13. Where are the guns typically kept or stored at home? (Select all that apply)
    - o Within the living spaces (including bedroom)
    - o Basement
    - o Attics
    - o Garage/Shed
    - o Patio/porch/deck
14. Are there any other places, other than your home, where you keep a gun (example: Your car, work)?
    - o Yes
    - o No

If yes, please list where here:

____

15. What type(s) of gun(s) do you have at home? (Select all that apply)
    - o Handguns (including revolvers and pistols)
    - o Rifles
    - o Shotguns
    - o Assault/Military-style weapons (example AR 15)
    - o Other, please list other gun types here__________
16. What is the primary purpose of your gun(s)?
    - o Safety/Protection
    - o Recreational (sport, hunting, or shooting range)
    - o Job: Armed forces, Law enforcement, Security
    - o Display/Decoration
    - o Family Heirloom
    - o Other purpose for gun, please state other purpose here______
17. What kind of gun safe storage device do you currently use for your gun(s)? (Select all that apply)
    - o None
    - o Trigger lock
    - o Cable lock
    - o Gun lockbox
    - o Gun safe
    - o Other, please list other type of safety storage device here__________
18. How often do you use these gun safe storage devices (Trigger lock, Cable lock, Lock box, Gun safe)?
    - o Most of the time
    - o Sometimes

- o Rarely
- o Always
- o Never

19. Are the gun(s) currently stored in your home locked? (Such as with a locked cabinet, locked box, with a trigger lock or cable gun lock)
    - o Yes, all are stored locked
    - o Yes, some are stored locked
    - o None of the guns are stored locked
    - o I don't know
20. Are there any gun(s) in your home currently stored loaded with bullets/ammunition?
    - o Yes
    - o No
21. Are the bullets/ammunition kept in a location separate from the gun?
    - o Yes
    - o No
22. Do you or anyone else ever take the gun out of the house (car, purse, etc.)?
    - o Yes
    - o No
23. If your child is in a time of crisis, have you ever stored your firearms outside of the home?
    - o Yes
    - o No

    If Yes, where have they been stored? Please state here_________
24. We are giving free firearm safe storage devices today (gun lock boxes and trigger locks). Would you like one?
    - o Yes
    - o No

    If yes please select the type of device or devices that you prefer?
    - o Gun lockbox (combination code)
    - o Trigger lock
    - o Both gun lockbox (combination code) plus trigger lock
25. What's the main reason you want a gun safety device?
    - o To keep people in my home or who visit my home safe
    - o To keep gun from being stolen
    - o To store other valuables (not gun)
    - o I don't want a gun safety device
    - o Other, please list other reason here____________
26. Do you plan on immediately using this safety device to store your gun(s)?
    - o Yes
    - o No
    - o Not sure
    - o I am not taking a safety device today

Caregiver feedback (all participants)

On a scale of 1 to 5, 1 being strongly disagree and 5 being strongly agree with the following statements, please select your score

27. I found the gun safety education shared today useful and/or helpful in keeping my home safe.
    - 1- Strongly disagree
    - 2- Disagree
    - 3- Neutral
    - 4-Agree
    - 5-Strongly agree
28. I believe the ED is an appropriate place to discuss gun safety.
    - 1- Strongly disagree
    - 2- Disagree
    - 3- Neutral
    - 4-Agree
    - 5-Strongly agree
29. After receiving gun safety education here today, will you ask about guns in the home before your child goes for a playdate or your teenager visits other people's homes?
    - 1- Strongly disagree
    - 2- Disagree
    - 3- Neutral
    - 4-Agree
    - 5-Strongly agree
30. To improve our process of providing gun safety education in the emergency department: What additional questions do you have or what information would you like to know about gun safety? Please state here____
31. We would like to follow up on the safety education and/or safety device given here in the ED approximately 1-2weeks from the ED visit. Please let us know your preferred method of being contacted. We will also be emailing gift cards for participation in follow up surveys.
    - Email
    - Text
    - Phone call

.

Caregiver 1 week Follow-up Survey Tool

Please fill out our study survey below. In exchange for the time you have taken to complete this survey, you will be emailed an electronic gift card. Thank you!

Caregiver feedback (all participants)

On a scale of 1 to 5, 1 being strongly disagree and 5 being strongly agree with the following statements, please select your score

1. I found the gun safety education shared in the emergency department useful and/or helpful in keeping my home safe.
   - 1- Strongly disagree
   - 2- Disagree
   - 3- Neutral
   - 4-Agree
   - 5-Strongly agree
2. I believe the emergency department is an appropriate place to discuss gun safety.
   - 1- Strongly disagree
   - 2- Disagree
   - 3- Neutral
   - 4-Agree
   - 5-Strongly agree
3. After receiving gun safety education in the emergency department, will you ask about guns in the home before your child goes for a playdate or your teenager visits other people's homes?
   - 1- Strongly disagree
   - 2- Disagree
   - 3- Neutral
   - 4-Agree
   - 5-Strongly agree

4. Have you shared any of the gun safety information you received with others: Your community, family, or friends?
   - Yes
   - No
5. Since your child's ER visit, before your child goes for a playdate or before your teenager visits a new place: Have you asked about guns being present (example: friend's or relatives' home)?
   - Yes
   - No

If no, why not?

- My child or teen has not visited other homes since the ER visit
- I'm too uncomfortable to ask
- It was a family member or someone I trust
- My child doesn't go on play dates or to other people's homes

- o Other, please state other reason here____________

Questions for Gunowner Caregivers Only

6. Have you removed any guns from the home since your emergency department visit either temporarily or permanently?
   - o Yes
   - o No

If yes, what were the reasons for removing the gun from your home? (Select all that apply)

- o Concern of someone in the home being able to use the gun to harm themselves or others
- o To keep kids in home safe
- o To keep adults in home safe
- o Other, please list other reasons here

7. What kind of gun safe storage device do you currently use for your gun(s)? (Select all that apply)
   - o None
   - o Trigger lock
   - o Cable lock
   - o Gun lockbox
   - o Gun safe
   - o Other, please list other type of safe storage device here:_____________
8. How often do you use these gun safe storage devices (Trigger lock, Cable lock, Lock box, Gun safe)?
   - o Most of the time
   - o Sometimes
   - o Rarely
   - o Always
   - o Never
9. Are the gun(s) currently stored in your home locked? (Such as with a locked cabinet, locked box, with a trigger lock or cable gun lock)
   - o Yes, all are stored locked
   - o Yes, some are stored locked
   - o None of the guns are stored locked
   - o I don't know
10. Are there any gun(s) in your home currently stored loaded with bullets/ammunition?
    - o Yes
    - o No
11. Are the bullets/ammunition kept in a location separate from the gun?
    - o Yes
    - o No

Question for Gunowner Caregivers that Took Study Device Only

12. Which device did you take from the ED?
    - o Lockbox only

- Trigger lock only
- Both a lockbox and trigger lock
